# The effect of a parenteral-to-oral conversion program for high-bioavailability antibiotics use

**DOI:** 10.1017/ash.2022.60

**Published:** 2022-05-16

**Authors:** Wooyoung Jang, Bongyoung Kim

## Abstract

**Background:** Appropriate conversion of antibiotics from parenteral to the oral route can lower the risk of catheter-associated infections, reduce medical costs, and shorten hospitalization. We investigated the effect of a parenteral-to-oral conversion program on high-bioavailability antibiotics conducted by medical students and the changes in perceptions of oral antibiotics after participating in the program. **Methods:** The parenteral-to-oral conversion program was implemented as a core clinical practice course for the fifth-year medical students in 2021 at the infectious diseases department in an affiliated hospital of a medical school in Korea. Half of the students in this class participated in the program from January to October 2021. An evaluation of the possibility of oral conversion was performed for parenterally administered, high oral-bioavailability antibiotics including ciprofloxacin, levofloxacin, moxifloxacin, metronidazole, linezolid, and trimethoprim–sulfamethoxazole. These agents are prescribed in the departments of pulmonology, gastroenterology, general surgery, and neurology. The medical students reviewed medical records for the patients treated with those antibiotics and wrote a recommendation for oral conversion for the cases with “possible oral conversion” after an infectious disease specialist confirmed their assessments. The cases without administration of any oral drugs or with the duration of parenteral antibiotic use of <3 days were excluded from the evaluation. The following cases were considered as “impossible oral conversion” and were excluded from the intervention: (1) admitted to the ICUs, (2) admitted to the protected isolation rooms, (3) difficult to take oral medication, (4) risk of insufficient medication absorption, e) bone and joint infections, (5) fever within 24 hours, (6) insufficient response to antibiotic therapy, and (7) recommended to use intravenous antibiotics by consultation with an infectious disease specialist. Furthermore, a survey was conducted on the perception of oral antibiotics in medical students before and after clinical practice to evaluate the educational effect of this program. **Results:** In total, 923 cases were reviewed, and 190 (20.6%) of 923 antibiotics prescriptions with high oral bioavailability were found to be administered parenterally even though they could be converted oral administration. Among these 190 antibiotics prescriptions, 46 (24.2%) were changed via a written proposal within 48 hours, 83 (43.7%) proposed changes were declined, and 61 (32.1%) antibiotics prescriptions were discontinued within 48 hours. Through this program, students have gained a better perception of oral antibiotics. **Conclusions:** This parenteral-to-oral conversion program showed a 24.2% acceptance rate of oral antibiotics conversions in the hospital, and it had significant educational effects on medical students regarding an appropriate perception of oral antibiotics.

Funding: None

Disclosures: None

